# Pharmacokinetics and diuretic effect of furosemide after single intravenous, oral tablet, and newly developed oral disintegrating film administration in healthy beagle dogs

**DOI:** 10.1186/s12917-021-02998-4

**Published:** 2021-09-06

**Authors:** Suk-Kyu Koh, Jong-Woo Jeong, Seo-In Choi, Rae Man Kim, Tae-Sung Koo, Kwan Hyung Cho, Kyoung-Won Seo

**Affiliations:** 1 Department of Veterinary Internal Medicine, College of Veterinary Medicine, Chungnam National University, 34134 Daejeon, Republic of Korea; 2 Graduate School of New Drug Discovery and Development, Chungnam National University, 34134 Daejeon, Republic of Korea; 3 College of Pharmacy and Inje Institute of Pharmaceutical Sciences and Research, Inje University, 50834 Gimhae, Republic of Korea; 4 Department of Veterinary Internal Medicine, College of Veterinary Medicine, Seoul National University, 08826 Seoul, Republic of Korea

**Keywords:** Canine, Furosemide, Loop diuretic, Oral film, Tablet, Conventional administration, Congestive heart failure, Diuresis, Noncompliance

## Abstract

**Background:**

Furosemide, a diuretic that acts on the loop of Henle, is commonly used to treat congestive heart failure in veterinary medicine. Some owners have difficulty in administering oral tablet medication to animal patients, which leads to noncompliance, especially during long-term administration. Oral disintegrating film (ODF) has the advantages of easy administration via a non-invasive route, rapid dissolution, and low suffocating risk. The objective of this study was to research the pharmacokinetic (PK) profiles and diuretic effect of furosemide after intravenous (IV), orally uncoated tablet (OUT), and newly developed ODF administration in healthy beagle dogs. In this study, a furosemide-loaded ODF (FS-ODF) formulation was developed and five beagle dogs were administered a single dose (2 mg/kg) of furosemide via each route using a cross-over design.

**Results:**

The most suitable film-forming agent was sodium alginate; thus, this was used to develop an ODF for easy drug administration. No significant differences were detected in the PK profiles between OUT and FS-ODF. In the blood profiles, the concentration of total protein was significantly increased compared to the baseline (0 h), whereas no significant difference was detected in the concentration of creatinine and hematocrit compared to the baseline. FS-ODF resulted in a similar hourly urinary output to OUT during the initial 2 h after administration. The urine specific gravity was significantly decreased compared to the baseline in each group. The peak times of urine electrolyte (sodium and chloride) excretion per hour were 1 h (IV), 2 h (OUT), and 2 h (FS-ODF).

**Conclusions:**

These results suggest that the PK/PD of furosemide after administration of newly developed FS-ODF are similar to those of OUT in healthy dogs. Therefore, the ODF formulation has the benefits of ease and convenience, which would be helpful to owners of companion animals, such as small dogs (< 10 kg), for the management of congestive heart failure.

## Background

Furosemide, a diuretic commonly used in veterinary and human medicine, is recommended as first-line therapy in the management of congestive heart failure (CHF) [1, 2]. It is used in animals for the treatment of pulmonary edema, udder edema, hypercalciuric nephropathy, uremia, and hypertension. Additionally, it reduces the incidence of sterile hemorrhagic cystitis associated with cyclophosphamide administration in dogs and is used as adjunctive therapy in hyperkalemia [2–10]. The pharmacokinetics and pharmacodynamics properties of loop diuretics, including furosemide after IV and oral (PO) administration, have been well investigated in both human and veterinary medicine [5, 8, 11–14].

Furosemide decreases the absorption of electrolytes in the luminal surface of the thick ascending loop of Henle via the deactivation of the Na^+^-K^+^-2Cl^−^ cotransporter. Thus, this drug increases the renal excretion of sodium, potassium, chloride, and water [8]. Additionally, furosemide causes renal venodilation, increases glomerular filtration rate, increases renal blood flow, and decreases peripheral resistance. Furthermore, furosemide activates the renin-angiotensin-aldosterone-system and sympathetic nervous system [15–17]. Although furosemide increases renin secretion, owing to its effects on the nephron, increases in sodium and water retention do not occur [8].

Conventional routes of furosemide administration are intravenous (IV), intramuscular (IM), subcutaneous (SC), or oral (PO). Unless an acute heart failure event occurs, PO administration is indicated in most cases that require long-term use in stable patients [8, 9, 18]. The onset time (IV, 5 min), elimination half-life (IV, 1–1.5 h), duration (IV, 3–6 h), and peak urine output (IV and SC, 1 h; PO, 2 h) of furosemide have been shown in previous experiments in dogs [8].

However, some owners have difficulty administering oral tablet medications, which leads to noncompliance, especially with drugs that require long-term administration. Particularly with cats, it is often difficult to administer pills or capsules. Beyond the conventional administration routes and formulations, studies have been conducted to identify options with similar efficacy that can be easily administered. For human cardiac disease, these studies have led to the development of the sildenafil citrate sublingual tablet to treat pulmonary arterial hypertension, nitroglycerin ointment to treat CHF, transdermal tulobuterol patch for bronchodilation, and transdermal beta-blocker (bisoprolol) patch to reduce postoperative atrial fibrillation [19–22].

The oral disintegrating film (ODF) offers several benefits especially in children, including easy administration via a non-invasive route, fast dissolution, and no risk of choking [23], that also can be utilized in veterinary medicine. Moreover, sublingual and oral furosemide administration differ in pharmacokinetic and pharmacodynamic results in humans [11]; the sublingual route may provide therapeutic advantages over the oral route, especially in patients with CHF. In veterinary medicine, there is a study on the diuretic effect of furosemide according to the IV, SC, PO, and constant rate infusion (CRI) routes in dogs [18]. The study on furosemide administration using sublingual bioadhesive film showed *ex vivo* mucoadhesion using the buccal mucosa and permeability using the tongue excised from slaughtered pigs [24]. And no alternative route of administration of furosemide has been reported, except for a study that the therapeutic effect of the transdermal application of furosemide to cats is negligible [7]. However, there are no announced studies on alternative administration routes in dogs. Despite there is a need for formulations that can be safely administered to and easily absorbed by animals, ODFs for use in dogs (especially small dogs) and cats have not been developed.

 The first objective of this study was to develop a suitable film applicable containing furosemide, which provide advantages over conventional administration routes in veterinary medicine. The second objective of this study was to compare the PKs and diuretic effect of furosemide after newly developed furosemide-loaded ODF (FS-ODF), conventional oral commercial tablet, and IV administration. We hypothesized that the newly developed FS-ODF would have similar drug when compared to oral tablets when administered at the same dose. .

## Results

Based on previous studies, film-forming agent and other excipients, including the plasticizer, solubilizer, disintegrant, sweetener, and solvent, were investigated for the most suitable agent and sodium alginate was selected to form the ODF.

**Fig. 1:**
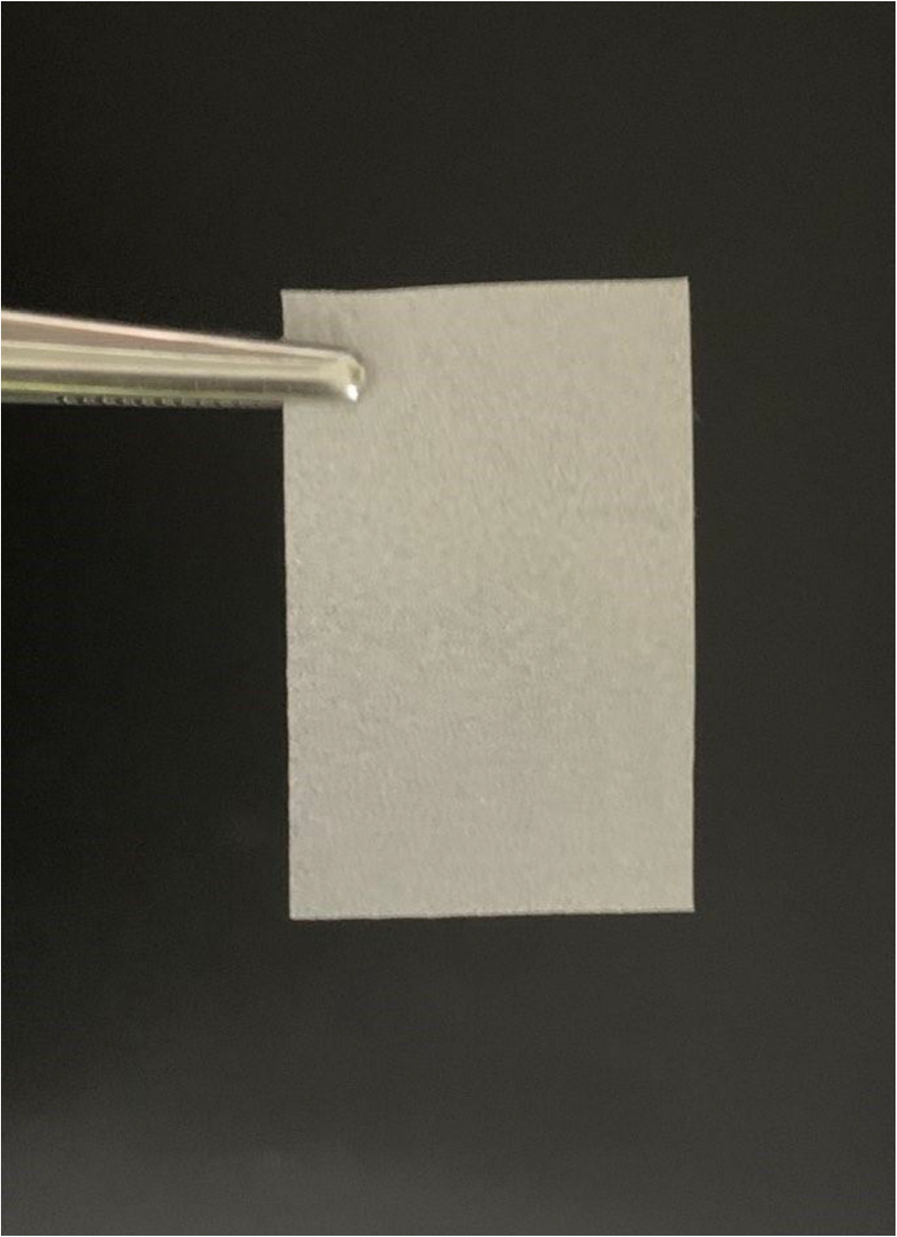
Representative appearance of furosemide-loaded orally disintegrating film. Legend: No content

The FS-ODF was prepared using the formulation shown in Table 1. Each FS-ODF had a size of 2 × 3 cm^2^ and weight of each film is 50 mg. This film had a homogeneous surface with some turbidity (Fig. 1). The mean weight of the film and drug content were 51.75 ± 2.25 mg and 97.63 % ± 1.87 %, respectively The disintegration time was 33.5 ± 4.5 s in water and the bending count, which represents tensile strength, was 4.4 ± 0.5.

**Table 1:** Formulation of FS-ODF

Purpose of use	Formulation (mg)	
Acitive ingredient	Furosemide	20
Film forming agent	Sodium alginate	14
Plasticizer	Glycerin	4
	PEG 400	4
Solubilizer	Tween 80	1
Sweetener	D-Sorbitol	1
Disintegrant	Crospovidone	6
Total weight (mg)		50

**Fig. 2:**
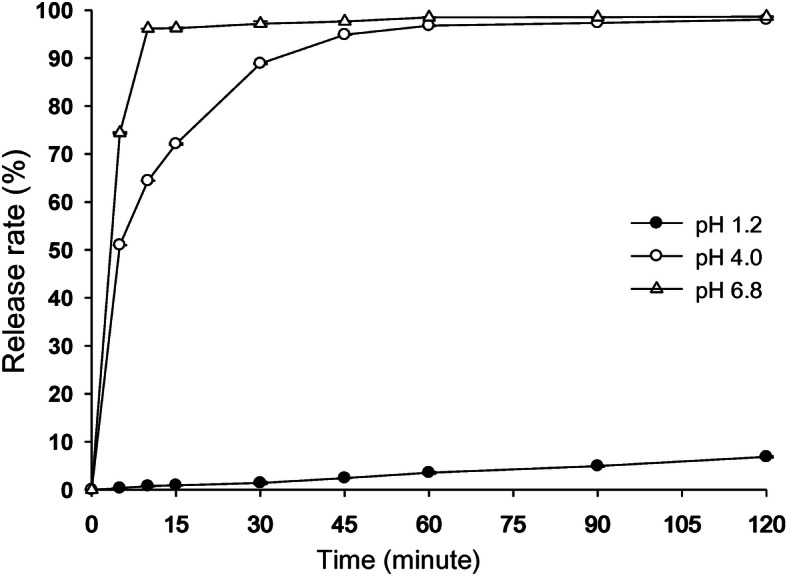
Dissolution profiles of furosemide-loaded orally disintegrating film at pH 1.2, pH 4.0, pH 6.8. (*n* = 4). Legend: No content

In the dissolution test, FS-ODF showed a pH-dependent curve (Fig. 2). The dissolution rate at pH 4.0 (96.75 %) and pH 6.8 (98.48 %) was higher than that at pH 1.2 (3.54 %) after 60 min. The dissolution rate at pH 1.2 was less than 10 % after 120 min. However, FS-ODF reached complete dissolution of more than 95 % at pH 4.0 and pH 6.8 within 60 min.

The dose of furosemide via all three routes was well-tolerated in all dogs. The vital signs on physical examination and behavior did not change during the study period.

**Fig. 3:**
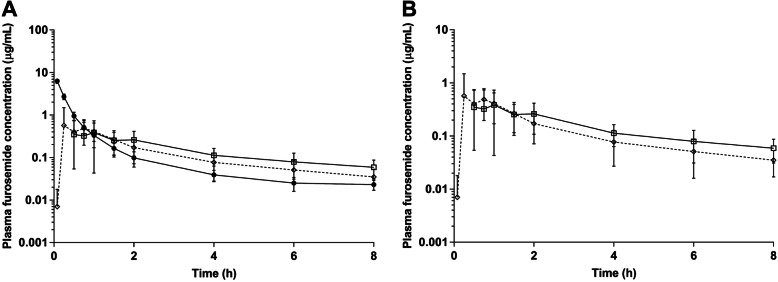
Mean plasma furosemide concentration for each group. Legend: **A** Comparison of time course change in plasma concentration following 2 mg/kg furosemide administration in intravenously (IV), orally uncoated tablet (OUT) and furosemide-loaded orally disintegrating film (FS-ODF) group. **B** OUT and FS-ODF are represented by a line graph in (**A**). IV, (●); OUT, (□); FS-ODF, (◇)

PK data of furosemide after the three routes of administration are summarized in Table 2 and presented in Fig. 3. No significant differences were detected between the orally uncoated tablet (OUT) and FS-ODF. In our study, the PK results of plasma furosemide showed a mean C_max_ of 9.65 µg/mL (IV, C_0_), 0.61 µg/mL (OUT), and 0.81 µg/mL (FS-ODF), mean AUC_last_ of 2.67 µg·h/mL (IV), 1.16 µg·h/mL (OUT), and 1.11 µg·h/mL (FS-ODF), and mean T_1/2_ of 2.07 h (IV), 3.44 h (OUT), and 2.64 h (FS-ODF).

**Table 2:** Pharmacokinetic parameters of furosemide (2 mg/kg) based on route of administration in beagle dogs

Pharmacokinetic parameters	IV	OUT	FS-ODF	P OUT vs. FS-ODF
C_max_ (μg/mL)	9.65 ± 1.63 (10.35)	0.61 ± 0.31 (0.69)	0.81 ± 0.81 (0.58)	0.619
T_max_ (h)	0.00	1 (0.5-1.5)	0.75 (0.25-1)	0.130
T_1/2_ (h)	2.07 ± 0.76 (1.63)	3.44 ± 1.79 (2.86)	2.64 ± 0.75 (2.46)	0.602
MRT (h)	0.90 ± 0.25 (0.78)	5.08 ± 1.67 (4.13)	3.47 ± 0.76 (3.55)	0.128
AUC_inf_ (μg·h/mL)	2.74 ± 0.25 (2.60)	1.50 ± 0.73 (1.72)	1.25 ± 0.68 (1.34)	0.493
AUC_last_ (μg·h/mL)	2.67 ± 0.26 (2.56)	1.16 ± 0.46 (1.44)	1.11 ± 0.63 (1.08)	0.734
CL (mL/h/kg)	735.7 ± 63.84 (768.98)	-	-	-
V_ss_ (mL/kg)	667.5 ± 222.6 (597.82)	-	-	-
BA (%)	-	54.98 ± 26.51 (62.74)	45.73 ± 25.02 (48.82)	0.493

*C*_*max*_ maximum plasma concentration, C_max,IV_ = C_0_, *T*_*max*_ time at the maximum concentration, *T*_*1/2*_ elimination half-life, *MRT* mean residence time, *AUC*_*inf*_ area under the curve from time zero to time of infinity measurable concentration, *AUC*_*last*_ area under the curve from time zero to time of last measurable concentration, *BA* bioavailability, *CL* elimination clearance, *V*_*ss*_ volume of distribution at steady state, *IV* intravenous, *OUT* orally uncoated tablet, *FS-ODF* furosemide-loaded orally disintegrating film. Results are presented as mean ± SD (median). However, T_max_, a categorical variable, expressed as median and range. (*), values are significantly different (*P* < 0.05); (**) values are significantly different (*P* < 0.01)

Plasma concentration of total protein was significantly increased 4 h after IV, 1–4 h after OUT, and 2–4 h after FS-ODF administration compared to baseline. However, the plasma blood urea nitrogen (BUN) concentration was significantly increased 1–2 h after IV and decreased 1 h and 4–8 h after FS-ODF administration compared to baseline. The plasma concentrations of creatinine and hematocrit were not significantly different compared to the baseline at any time point (Table 3). Moreover, the serum potassium concentration was significantly decreased 1–6 h after IV and OUT and 1–8 h after FS-ODF administration, and the serum chloride concentration was significantly decreased 2 h after IV and OUT administration. Compared to that at baseline, the serum sodium concentration did not change significantly over time (Table 4). Additionally, indirect systolic blood pressure was not different among application routes nor time points.

**Table 3:** Hematocrit and biochemistry profile following single dose of furosemide (2 mg/kg) administration

		Baseline	Time (h)				
			1	2	4	6	8
Hematocrit(%)	IV	40.46 ± 9.43	41.36 ± 5.43	38.52 ± 6.84	39.06 ± 7.43	39.02 ± 6.01	40.22 ± 5.35
	OUT	38.98 ± 4.18	39.10 ± 3.16	38.52 ± 3.50	38.64 ± 3.75	38.72 ± 4.04	40.12 ± 3.94
	FS-ODF	41.94 ± 5.19	41.02 ± 6.17	40.20 ± 6.48	41.44 ± 4.21	41.58 ± 6.30	40.54 ± 7.81
BUN (mg/dL)	IV	17.10 ± 6.33	17.50 ± 5.60^**^	17.20 ± 5.57^**^	16.72 ± 5.25	16.20 ± 5.21	15.94 ± 5.23
	OUT	21.84 ± 9.99	20.82 ± 9.12^**^	20.22 ± 8.99	19.04 ± 7.96	17.52 ± 6.25	16.60 ± 5.83
	FS-ODF	18.96 ± 6.39	18.68 ± 6.15^**^	18.16 ± 6.10	17.30 ± 5.90^**^	16.14 ± 5.28^*^	15.41 ± 4.72^*^
Cr (mg/dL)	IV	0.72 ± 0.24	0.68 ± 0.19	0.68 ± 0.24	0.68 ± 0.19	0.72 ± 0.13	0.68 ± 0.23
	OUT	0.72 ± 0.29	0.70 ± 0.25	0.68 ± 0.23	0.66 ± 0.23	0.68 ± 0.16	0.66 ± 0.18
	FS-ODF	0.72 ± 0.13	0.74 ± 0.19	0.68 ± 0.22	0.70 ± 0.19	0.72 ± 0.19	0.70 ± 0.20
Total protein (mg/dL)	IV	5.90 ± 0.36	6.32 ± 0.29**	6.18 ± 0.36**	6.10 ± 0.30*	6.04 ± 0.25	6.04 ± 0.34*
	OUT	5.74 ± 0.29	6.02 ± 0.25**	6.10 ± 0.38**	6.10 ± 0.31*	5.94 ± 0.34	6.00 ± 0.29*
	FS-ODF	6.06 ± 0.18	6.30 ± 0.10**	6.38 ± 0.18**	6.26 ± 0.17*	6.30 ± 0.20	6.34 ± 0.05*

Data are described as mean ± SD values. *IV* intravenous, *OUT* orally uncoated tablet, *FS-ODF* furosemide-loaded orally disintegrating film, *BUN* Blood urea nitrogen, *Cr* Creatinine. The *p*-values are significantly different (*p < 0.05, **p < 0.01) compared to the baseline

**Table 4:** Serum electrolyte concentration following furosemide (2 mg/kg) administration in beagle dogs

		Baseline	Time (h)				
			1	2	4	6	8
Serum sodium excretion (mmol)	IV	151.26 ± 4.06	148.14 ± 1.46	148.04 ± 1.13	150.60 ± 2.10	147.74 ± 4.79	148.52 ± 1.30
	OUT	150.34 ± 2.62	153.56 ± 7.59	150.14 ± 1.48	149.12 ± 1.08	151.92 ± 5.29	149.84 ± 1.44
	FS-ODF	150.94 ± 2.97	155.20 ± 9.26	150.06 ± 2.12	148.98 ± 6.06	153.46 ± 9.29	148.56 ± 1.09
Serum potassium excretion (mmol)	IV	4.48 ± 0.15	4.19 ± 0.17^*^	3.97 ± 0.29^**^	4.12 ± 0.29^**^	4.04 ± 0.31^**^	4.15 ± 0.42
	OUT	4.51 ± 0.34	4.43 ± 0.41^*^	3.97 ± 0.14^**^	4.05 ± 0.23^**^	4.04 ± 0.28^**^	4.05 ± 0.27
	FS-ODF	4.43 ± 0.32	4.08 ± 0.36^**^	3.92 ± 0.28^**^	4.09 ± 0.37^**^	4.13 ± 0.41^*^	4.05 ± 0.23^*^
Serum chloride excretion (mmol)	IV	110.66 ± 4.20	106.46 ± 1.41	105.96 ± 1.33^*^	107.64 ± 2.01	104.34 ± 4.25	106.36 ± 0.80
	OUT	110.28 ± 3.38	111.56 ± 8.26	107.52 ± 1.61^*^	105.88 ± 0.58	107.76 ± 3.43	106.50 ± 1.35
	FS-ODF	110.22 ± 3.30	112.74 ± 7.69	106.84 ± 1.82	106.04 ± 4.56	109.96 ± 7.89	106.20 ± 1.08

Results are presented as mean ± SD. *IV* intravenous, *OUT* orally uncoated tablet, *FS-ODF* furosemide-loaded orally disintegrating film. The *p*-values are significantly different (*p < 0.05, **p < 0.01) compared to the baseline

The hourly urine output (HUO) and accumulated urinary output are shown in Table 5. The HUO in the 1–2 h after administration was increased significantly compared to the baseline in each group. The HUO peak of IV administration was high and early, whereas that of OUT and FS-ODF administration was broader and appeared later. The HUO was increased 1 h after IV administration, then returned to the baseline level after 4 h. The HUO for OUT and FS-ODF was increased 1–2 h after administration and returned to the baseline level after 6 h. The mean duration of diuresis until the return to baseline levels after IV, OUT, and FS-ODF administration was 4.1 h, 5.72 h, and 5.24 h, respectively. Similar value of HUO were observed between OUT and FS-ODF during initial 2 h after administration. The total urine output was 24.65 mL/kg, 30.9 mL/kg, and 25.4 mL/kg during 8 h after IV, OUT, and FS-ODF administration, respectively. -Urine-specific gravity (USG) and urine electrolyte excretion per hour (UEEH) are shown in Table 6. The USG was significantly decreased after administration in all groups but returned to the baseline after 8 h. The UEEH was significantly increased 1–2 h after administration in all groups. The peak times of sodium and chloride UEEH were 1 h, 2 h, and 2 h, whereas those of potassium UEEH were 1 h, 1 h, and 2 h, after IV, OUT, and FS-ODF administration, respectively.

**Table 5:** Hourly urine output and cumulative urine output after furosemide administration via different routes

		Baseline	Time (h)				
			1	2	4	6	8
Hourly urinary output (ml/h)	IV	8.45 ± 2.82	147.20 ± 34.05^**^	74.60 ± 15.61^**^	9.70 ± 3.93	7.10 ± 3.11	6.20 ± 3.68
	OUT	16.40 ± 22.28	88.80 ± 94.10^**^	128.80 ± 51.09^**^	34.30 ± 16.90	13.50 ± 5.51	6.60 ± 4.59
	FS-ODF	12.66 ± 6.97	71.40 ± 41.93^*^	121.20 ± 61.49^*^	18.0 ± 8.96	9.40 ± 4.48	6.70 ± 2.31
Accumulatedurinary output (ml)	IV	13.0 ± 4.53	160.20 ± 37.16^**^	234.80 ± 47.07^**^	254.20 ± 51.28^**^	268.4 ± 57.05^**^	288.6 ± 55.76^**^
	OUT	26.20 ± 32.22	115.0 ± 124.80^**^	243.80 ± 151.63^**^	312.40 ± 139.36^**^	339.4 ± 135.60^**^	359.8 ± 134.75^**^
	FS-ODF	27.0 ± 16.90	98.40 ± 41.97^*^	219.60 ± 89.41^**^	255.60 ± 91.68^**^	274.40 ± 87.68^**^	293.60 ± 99.60^**^

Data are described as mean ± SD values. *IV* intravenous, *OUT* orally uncoated tablet, *FS-ODF* furosemide-loaded orally disintegrating. The *p*-values are significantly different (*p < 0.05, **p < 0.01) compared to the baseline

**Table 6:** Urine-specific gravity and urine electrolyte excretion after furosemide (2 mg/kg) administration

		Baseline	Time (h)				
			1	2	4	6	8
Urine-specific gravity	IV	1.040 ± 0.010	1.012 ± 0.002^**^	1.007 ± 0.001^**^	1.011 ± 0.002^**^	1.020 ± 0.004^*^	1.030 ± 0.007
	OUT	1.039 ± 0.009	1.032 ± 0.015^**^	1.012 ± 0.006^**^	1.010 ± 0.003^**^	1.017 ± 0.006^*^	1.022 ± 0.007
	FS-ODF	1.045 ± 0.007	1.022 ± 0.013^*^	1.009 ± 0.002^**^	1.014 ± 0.005^**^	1.020 ± 0.010^*^	1.025 ± 0.009^*^
Urine sodium excretion per hour (mmol/h)	IV	0.64 ± 0.56	17.75 ± 7.21^**^	8.70 ± 3.52^**^	0.95 ± 0.47	0.52 ± 0.21	0.35 ± 0.21
	OUT	0.95 ± 1.05	11.13 ± 11.67^**^	18.88 ± 10.01^**^	3.77 ± 1.77	1.22 ± 0.52	0.44 ± 0.22
	FS-ODF	0.93 ± 0.56	8.63 ± 4.63^*^	17.18 ± 12.01^*^	1.96 ± 0.94	0.78 ± 0.24	0.48 ± 0.11
Urine potassium excretion per hour (mmol/h)	IV	0.31 ± 0.16	3.51 ± 1.17^**^	1.11 ± 0.32^**^	0.21 ± 0.10	0.16 ± 0.06	0.16 ± 0.11
	OUT	0.92 ± 1.12	4.48 ± 4.48^**^	2.46 ± 1.24^**^	0.63 ± 0.26	0.33 ± 0.18	0.14 ± 0.06
	FS-ODF	0.79 ± 0.53	2.40 ± 0.67^*^	2.70 ± 1.38^*^	0.41 ± 0.21	0.24 ± 0.13	0.16 ± 0.06
Urine chloride excretion per hour (mmol/h)	IV	0.43 ± 0.11	11.43 ± 3.04^**^	5.72 ± 1.33^**^	0.72 ± 0.33	0.32 ± 0.12	0.19 ± 0.08^**^
	OUT	1.04 ± 1.29	8.48 ± 9.14^**^	10.29 ± 4.41^**^	2.63 ± 1.36	0.84 ± 0.38	0.25 ± 0.09^**^
	FS-ODF	0.76 ± 0.47	4.89 ± 2.62^*^	9.60 ± 5.21^*^	1.39 ± 0.62	0.53 ± 0.23	0.30 ±0.09

Data are described as mean ± SD values. *IV* intravenous, *OUT* orally uncoated tablet, *FS-ODF* furosemide-loaded orally disintegrating film. The *p*-values are significantly different (*p < 0.05, **p < 0.01) compared to the baseline

## Discussion

To the authors’ knowledge, this study is the first to compare the PK parameters and diuretic effect of IV, OUT and ODF administration of furosemide in dogs. Among the conventional routes of furosemide administration, PO administration is the most common route for long-term management in veterinary patients with CHF [8, 9]. However, in the case of home care, oral tablet administration may be limited by low patient cooperation and it is difficult to use other traditional administration routes, such as IM, SC, and IV. For the reasons mentioned earlier, we studied a useful alternative routes that could be applied to veterinary medicine.

 Usually, ODFs disintegrate within 1 min in the oral cavity owing to contact with the saliva, which results in rapid absorption. Furthermore, when administered via buccal or sublingual routes, there are advantages of high permeability and bypassing first-pass metabolism [23]. In this study, the ODF dissolved in the mouths of dogs quickly without water, which indicates that the ODF formulation is beneficial with regard to rapid dissolution, absorption, and ease of drug administration in veterinary patients. Therefore, a low-dose FS-ODF formulation for small animals was developed.

Based on our research results, it was decided to use sodium alginate as film-forming agent containing furosemide. Then, 20 mg of furosemide (a high ratio; 40 %, w/w) was loaded per sheet, which was cut depending on the body weight of the dog to reach a dose of 2 mg/kg. These physical specifications were achieved for convenience of use in small animals, such as small dogs (< 10 kg) or cats [25]. The small and uniform particles of furosemide in the final preparation suspension enabled the homogeneous and desirable film surface and might have reduced weight variation and content loss [26]. The formulation was optimized, with a disintegration time of less than 40 s, owing to the presence of crospovidone as a disintegrant. Rapid disintegration to small particles in the mouth is an essential property to ensure the good usability of a film [27]. Moreover, the bending count was in an acceptable range and showed the flexibility of the film. Overall, FS-ODF had suitable film properties in terms of appearance, drug content, disintegration time, and tensile strength.

In the dissolution test, FS-ODF showed a pH-dependent curve because furosemide has a highly pH-dependent solubility. Furosemide is a weak acid (pKa = 3.48) with a carboxylic acid functional group and its aqueous solubility increases as the pH increases from 0.18 mg/mL (pH 2.3) to 13.36 mg/mL (pH 10.0) [28]. Thus, in this study, the dissolution rate was in the order of pH 6.8 > pH 4.0 > > pH 1.2 within 60 min. FS-ODF showed immediate-release on the curve (Fig. 2), with high dissolution rates at pH 4.0 and pH 6.8 owing to the rapid disintegration time of less than 1 min. Therefore, FS-ODF was sufficient, in terms of disintegration time and dissolution rate, for administration to small animals.

In dogs and humans, the diuretic effect caused by furosemide continues for 3 h after IV injection. The concentration of furosemide initially exceeds the number of Na^+^-K^+^-2Cl^−^ cotransporters in the loop of Henle after IV administration and are excreted in the urine. Thereafter, the furosemide concentration declines under therapeutic concentrations [29–31].

Additionally, the bioavailability of furosemide is different depending on the administration method. For example, when administered orally, the first-pass effect, related to the extent of gastrointestinal absorption and metabolism, plays a major role in the bioavailability of furosemide. Also, the first-pass effect varies by species and individual. The diuretic effect of furosemide after PO administration has a slower onset than that after IV and SC administration [32]. Furthermore, the bioavailability of furosemide is approximately 77 % in dogs and 60–65 % in humans after PO administration [8, 13, 29]. The diuretic effect of furosemide continues 1–2 h after IV injection. Moreover, the absorption of furosemide is influenced by the pH and the existence of binding proteins in the stomach [18, 33, 34]. Although furosemide is slowly absorbed in the small intestine, it is absorbed rapidly in the stomach because of the acid environment. The absorption of furosemide in the gastrointestinal tract of patients with CHF may be lower than normal [35]. Generally, in patients with CHF, absorption delay may be the result of increased motility, reduced perfusion, or mucosal edema of intestine [36, 37].

The PKs of furosemide determine the therapeutic effect, entry rate of drug into the blood and urine, and onset and duration of effects, all of which are altered by the method of administration [18, 29, 30]. Both OUT and FS-ODF administration showed a slower onset and a longer elimination half-life than IV administration. Previous studies have shown a half-life of 0.5–1 h after IV administration, whereas the half-life has been reported as 30 min in the major disposition phase and approximately 7 h in the slow elimination phase after PO administration in dogs [5, 6, 38]. According to another PK/PD study in healthy beagle dogs, the half-life after PO administration in dogs is 3 h, which is similar to the results of the present study [39].

In the body, furosemide moves across the renal tubule from the plasma to the lumen of the nephron; thus, furosemide acting on henle’s loop of renal tubule should have a short duration of effects if its half-life is short [7]. These results appear to be owing to the initial high blood concentration and rapid elimination of furosemide after IV administration. In addition to the route of administration of the drug, it should also be considered that the half-life of drugs is affected by many factors, including illness status, age, and other physiological variables [40]. The present study demonstrates that the PKs after the administration of FS-ODF are similar to those after that of OUT in healthy dogs.

Furthermore, after IV administration, the HUO was significantly increased and peaked after 1 h, then decreased gradually to baseline levels. This result was similar to that of previous studies of the IV route [8]. The HUO of OUT and FS-ODF routes were significantly increased after 1–2 h and peaked after 2 h. Therefore, FS-ODF and OUT resulted in a similar HUO during the initial 2 h after administration.

The duration of diuresis (time until return to baseline level after furosemide administration) after OUT and FS-ODF administration was similar at 5 h. Additionally, UEEHs were increased during the initial 2 h after administration in all groups. Based on HUO, USG, and UEEH results, FS-ODF has equivalent aquaretic, natriuretic, kaliuretic, chloriuretic, and diuretic effects to conventional OUT.

This study has a few limitations, which may be overcome by further research. First, additional studies using different doses and repeating doses are needed because we used only a single dose of furosemide (2 mg/kg). Second, we only used five healthy intact beagle dogs. Additional studies should be conducted in more dog patients with CHF, renal failure, and delayed intestinal absorption. Third, since small animals have various body weight ranges, it is necessary to prove that they contain a homogeneous dose even when the film is cut to apply the recommended diuretic dose.

## Conclusions

In this study, we developed an ODF for small animals that showed similar PK parameters and diuretic effect when compared to OUT administration. Collectively, the results of this study suggest that the FS-ODF formulation can be used as an alternative to oral tablets in animal patients with heart failure and volume overload. Therefore, it would be helpful to owners of dogs that refuse conventional oral medications owing to the potential benefits of easy administration and convenient dosage form.

## Methods

### Animals

Five clinically healthy adult beagle dogs (three intact males and two intact females) dogs from a research colony at the College of Veterinary Medicine of Chungnam National University were included in this study. The animals were 5–9 years of age and weighed 10–13 kg. All dogs were normal on physical examination, including systemic blood pressure measurement with a Doppler device, and within normal ranges on complete blood count, serum biochemistry panel, serum electrolytes, and urinalysis. Animals were housed in cages and fed commercial food (SUNGBO Pet Healthcare, Seoul, South Korea) twice daily. Water was provided ad libitum. This study was approved by the Institutional Animal Care and Use Committee (IACUC) at Chungnam National University (approval number, CNU-01189). After the study, all dogs were adopted as companion animals.

### Preparation of FS-ODF

The FS-ODFs were prepared by a simple, easy solvent-casting method that required no special equipment [41]. For lab-scale preparation, as the batch size was 15 FS-ODFs, the amount of formulation for 15 FS-ODFs was weighed and used (Table 1). Furosemide and two plasticizers were dissolved in 4.5 mL of ethanol, heated in 80 °C chambers for 30 min. This solution was transferred to a 40 °C chamber to reach temperature equilibrium (Solution A). The other excipients, including the film-forming agent, solubilizer, and sweetener, were dissolved in 10.5 mL of water and the disintegrant was suspended. This aqueous solution was transferred to 40 °C chambers to reach temperature equilibrium (Solution B). Solution A was added to Solution B and the mixture was stirred at room temperature (22–24 °C) for 30 min to obtain a uniform suspension. This suspension was sonicated for 10 min for degassing. The amount of suspension equivalent to 10.60 films on an area basis was placed on a Petri dish (9 cm in diameter) and dried in 80 °C chambers for 40–45 min. The formed film was peeled off and cut to a predetermined size of 2 × 3 cm^2^.

### Characterization of FS-ODF

Disintegration time, bending count, weight and drug content tests for characterization of FS-ODF were each run 5 times.

### Disintegration Time

One FS-ODF was placed on a petri dish (9 cm diameter) with 15 mL of 37 ℃ water. The time to disintegrate completely was measured by visual observation [42]. The absence of any significant floating fragments was considered to be the completion of disintegration.

### Bending Count

The bending count measured by the number of times it took to split when the film was folded in half repeatedly with two fingers.

### Weight and Drug Content

The prepared FS-ODF was weighed using balance and transferred into 30 mL dilution solution (0.05 N NaOH solution) in 50 mL volumetric flask and dissolved with vortexing. Then, the dilution solution was added to the total volume. This solution of 2 mL was transferred to 100 mL volumetric flask and the dilution solution was added to the total volume. The final solution was filtered with syringe filter (0.45 μm) and the filtrate was analyzed by the below HPLC method.

### HPLC Condition

The HPLC analysis of furosemide in the samples was conducted using a Waters 2695 HPLC system (Waters, Milford, MA, USA) equipped with a UV-Vis detector (Waters 2487, Waters, Milford, MA, USA). Furosemide was analyzed using the reverse column with C18, 5 μm, 4.5 mm × 25 cm (Shiseido, Tokyo, Japan). The mobile phase consisted of water, tetrahydrofuran, and acetic acid (70:30:1, v/v/v). The HPLC analysis was performed with a flow rate of 1.2 mL/min. The injected volume of the sample was 20 µL, and UV detection was monitored at 272 nm [43]. Data acquisition and processing were carried out using the Waters LC Solution software.

### Dissolution Test

The dissolution test was performed using the USP [35] dissolution apparatus II at pH 1.2 (0.1 N HCl/NaCl buffer), pH 4.0 (acetate buffer), pH 6.8 (phosphate buffer) with the media volume of 900 mL at 37.0 ± 0.5 °C. The rotational speed was adjusted to 50 rpm. The prepared FS-ODF containing furosemide 20 mg in a sample was placed into a dissolution vessel with a sinker [44]. At each predetermined interval, the aliquot (5 mL) of the medium was collected and filtered through a membrane filter (pore size: 0.45 μm). The concentration of furosemide in the filtrate was determined using the above HPLC method.

### Study design

This study was performed using a single-dose, randomized, 3-way crossover design. Three methods of furosemide administration were used in each subject with at least 7-day washout period between each experiment. Animals were fasted for 12 h before the administration of furosemide and had a free access to water during the experiment.

Each (random) animals received a furosemide of 2 mg/kg via intravenous (IV), orally uncoated tablet (OUT), furosemide-loaded orally disintegrating film (FS-ODF) routes: (1) Furosemide 2 mg/kg IV (*n* = 5), (2) OUT formulation of furosemide 2 mg/kg orally (*n* = 5), (3) FS-ODF formulation 2 mg/kg orally (*n* = 5).

Injections and OUT formulation of furosemide were purchased from handok Pharm. Co. (Seoul, South Korea). For oral administration, furosemide OUT and FS-ODF formulation were cut according to body weight of each dog. Also, 22-Gauge IV catheter was used in the cephalic vein for the IV injections. All dogs received no other medications before the experiment and during the washout period.

### Sample collection

Blood samples were collected at 0 (serving as baseline), 5, 15, 30, 45, 60, 120, 240, 360, 480 min in each administration routes. All blood samples were obtained from the jugular vein with 23-Gauge 5mL syringe. These samples were immediately divided into heparinized tubes for biochemical test, plain tubes for electrolyte analysis and EDTA-K2 tube for hematocrit. Heparinized plasma and serum were separated after centrifugation at 3,000 x g for 15 min and stored at − 20 °C until analysis. A complete blood count, BUN, creatinine (Cr), plasma total protein and serum electrolytes were measured.

The urine samples were collected at 0 (serving as baseline), 1, 2, 4, 6, 8 h in each administration routes. In line with previous studies, the bladder was initially emptied 2 h before baseline and then collected in 2 h to calculate baseline hourly urine output (HUO) before furosemide administration. An indwelling 6–8 Fr balloon Foley catheter was placed into the urinary bladder at the initiation of each experiment and the bladder was emptied for urine volume measured at each time point. Urine sample was centrifuged (1500 × g, 10 min) and the supernatant was collected to verify urine-specific gravity and the concentrations of sodium, potassium and chloride.

Complete blood count was measured by ADVIA 2120i (SIEMENS corporation, Munich, Germany) and biochemistry were measured by Mindray BS-300 (Bio-Medical Electronics Co., Ltd, Shenzhen, China). Electrolyte concentrations in plasma and urine sample were measured by EasyLyte PLUS (MEDICA corporation, Bedford, USA).

### Quantification of furosemide in plasma

Plasma concentration of furosemide were determined by HPLC-MS/MS system. To prepare appropriate assay samples, plasma samples were processed with acetonitrile to induce precipitation of plasma protein. Firstly, 15 µL of plasma or calibrator sample were treated with 155 µL of 0.11 µg/mL ibuprofen (internal standard, IS) in acetonitrile. And then, sample mixtures were vigorously shaken by vortex mix for 15 min. Finally, sample mixtures were separated into supernatant and precipitant pellet by centrifugation at 17,600 g for 15 min. Standard samples were prepared by spiking of 5 µL working solution (as furosemide; 0, 30, 100, 300, 1,000, 3,000, 10,000, 30,000, and 100,000 ng/mL in acetonitrile) into 45 µL of blank plasma. For a sample analysis, 130 µL of supernatant were transferred into sample vials, and 5 µL of processed sample was directly injected into HPLC separation system.

Furosemide and IS were separated by Agilent 1100 HPLC system. Chromatographic separation was conducted on Agilent ZORBAX® phase phenyl column (5 μm, 2.1 × 50 mm) by 6 mM ammonium formate in 40 % acetonitrile solution.

After separation, both analytes were detected by triple quadrupole mass spectrometer (AB SCIEX API4000 QTRAP®). Furosemide and IS were monitored under electrospray ionization negative mode as multiple reaction monitoring, which mass to charge ratio of 329.08^−^ to 284.80^−^ for furosemide and 205.05^−^ to 161.10^−^ for IS, respectively.

Peak areas integration and quantification were automatically conducted by using Analyst software® (AB Sciex) 1.6.2. The retention times of furosemide and ibuprofen were 0.53 and 0.77 min, respectively. Concentration-response of furosemide was linear at ranges from 30 ng/mL to 10 µg/mL (r = 0.9971). The validation values, including the precision (coefficient of variance < 5.67 %), accuracy (relative error < 6.39 %) of the measurements, were within the acceptable ranges given by FDA guidelines.

### Pharmacokinetic analysis

Temporal profiles of furosemide in beagle were analyzed with via non-compartmental model using Phoenix WinNonlin 6.2. (Pharsight, USA). The following pharmacokinetic parameters were calculated and compared based on the route of administration: C_max_, half-life time, AUCs, CL, V_ss_, bioavailability (F). The elimination rate constant (k_e_) was determined by linear regression of the semi-log portion of the elimination phase. The elimination half-life (T_1/2_) was calculated by dividing 0.693 with k_e_. The area under the plasma concentration versus time curve from time zero to time of last measurable concentration (AUC_last_), the area under the plasma concentration versus time curve from time zero to infinity (AUC_inf_) and the area under the respective first moment time curve from time zero to infinity (AUMC_inf_) were calculated based on the linear-up and log-down method. To estimate the elimination clearance (CL) and volume of distribution at steady state (V_ss_), a moment analysis was conducted. In more detail, CL was gained by dividing administered dose with AUC_inf_. V_ss_ was obtained by multiply CL with MRT, which division of AUMC_inf_ by AUC_inf_. Fs of film and tablet groups were calculated from the dose normalized AUC_PO_/AUC_IV_, respectively.

### Statistical methods

All data were expressed as mean and standard deviation. Normality test was conducted with Kolmogorov-Smirnov test and pharmacokinetic parameters were an independent *t*-test. Because the study aimed to compare orally uncoated tablet with orally disintegrating film, only the differences between these two formulations of administration were statistically analyzed. Comparisons between baseline (0 h) and various time phases after drug administration were analyzed statistically using the paired *t*-test. A *P*-value < 0.05 was considered to be statistically significant. Statistical analysis was performed using SPSS 25 (SPSS Inc., Chicago, IL, USA).

## Data Availability

The data for figures, tables, and material used and analyzed during the current study are available from the corresponding author on reasonable request.

## Abbreviations

ODF: Oral disintegrating film
PK: Pharmacokinetics
IV: Intravenous
IM: Intramuscular
OUT: Irally uncoated tablet
FS-ODF: Furosemide loaded-orally disintegrating film
PO: Oral
CHF: Congestive heart failure
CRI: Constant-rate infusion
SC: Subcutaneous
C_max_: Maximum plasma concentration
C_0_: Maximum plasma concentration after IV
T_max_: Time at the maximum concentration
T_1/2_: Elimination half-life
AUC_inf_: Area under the curve from time zero to time of infinity measurable concentration
AUC_last_: Area under the curve from time zero to time of last measurable concentration
F: Fraction of oral dose absorbed (= bioavailability)
CL: The elimination clearance
V_ss_: Volume of distribution at steady state
MRT: Mean residence time
USG: Urine specific gravity
k_e_: Elimination rate constant
BUN: Blood urea nitrogen
Cr: Creatinine
HUO: Hourly urine output
UEEH: Urinary electrolytes excretion per hour
HPLC: High-performance liquid chromatography
HPLC-MS/MS: High-performance liquid chromatography-tandem mass spectrometry
